# Assessment of Useful Plants in the Catchment Area of the Proposed Ntabelanga Dam in the Eastern Cape Province, South Africa

**DOI:** 10.1155/2017/3763607

**Published:** 2017-07-30

**Authors:** Alfred Maroyi

**Affiliations:** Medicinal Plants and Economic Development (MPED) Research Center, Department of Botany, University of Fort Hare, Private Bag X1314, Alice 5700, South Africa

## Abstract

**Background:**

The developmental projects, particularly construction of dams, result in permanent changes of terrestrial ecosystems through inundation.

**Objective:**

The present study was undertaken aiming at documenting useful plant species in Ntabelanga dam catchment area that will be impacted by the construction of the proposed dam.

**Methods:**

A total of 55 randomly selected quadrats were used to assess plant species diversity and composition. Participatory rural appraisal (PRA) methods were used to identify useful plant species growing in the catchment area through interviews with 108 randomly selected participants.

**Results:**

A total of 197 plant species were recorded with 95 species (48.2%) utilized for various purposes. Use categories included ethnoveterinary and herbal medicines (46 species), food plants (37 species), construction timber and thatching (14 species), firewood (five species), browse, live fence, and ornamental (four species each), and brooms and crafts (two species).

**Conclusion:**

This study showed that plant species play an important role in the daily life and culture of local people. The construction of Ntabelanga dam is, therefore, associated with several positive and negative impacts on plant resources which are not fully integrated into current decision-making, largely because of lack of multistakeholder dialogue on the socioeconomic issues of such an important project.

## 1. Introduction

Local vegetation provides local people with a variety of services and goods that support human well-being and survival. According to Hamilton et al. [1], plant resources provide local communities with food, fuel, and medicine, as well as materials for construction and the manufacture of crafts and many other household products. Plant resources play a central role in the everyday lives of rural people in developing countries and marginalized areas, with their daily round of activities revolving around agriculture, the gathering of edible fruits, leafy vegetables, herbal medicines, fuelwood, the cooking and eating of largely plant-based food, and the construction of buildings and fences [1]. Local people are known to harbour important information on plant resources that are important for their livelihoods and such information is important for management strategies aimed at sustainable use and conservation of such plant resources [2]. For many local communities, the use of plant resources is a source of cultural identity, reflecting a deep and important body of knowledge about the environment, survival, and sustainable living known widely as traditional ecological knowledge (TEK) [3]. Berkes [4] defined TEK as a cumulative body of knowledge, practice, and belief, evolving by adaptive processes and handed down through generations by cultural transmission, about the relationship of living beings with one another and with their environment. TEK systems, therefore, incorporate means of communicating and transmitting environmental knowledge including information on the harvesting, processing, and sustainable use of plant resources, their seasons and cycles of production, their habitats and their use by other species [4]. The significance of TEK as an important vehicle for sustainable development was recognized in the Brundtland Commission's report, our common future [5], and at the Earth Summit in Rio de Janeiro in 1992 [6]. Traditional ecological knowledge has also been incorporated into the Agenda 21 document of the United Nations and International Convention on Biodiversity [6, 7] emphasizing the critical role of indigenous people and local communities and their knowledge in achieving sustainable environmental and natural resource management. Previous research in South Africa revealed that plant resources serve a number of functions including daily subsistence, income-generation, cash saving [8], safety nets during times of adversity [9, 10], and meeting spiritual and cultural needs [11]. It is within this context that an assessment of useful plants in the catchment area of the proposed Ntabelanga dam in the Eastern Cape province, South Africa, was carried out.

The Department of Water and Sanitation, South Africa, commissioned the construction of Ntabelanga dam on the Tsitsa river, an integrated multipurpose project aimed at rejuvenating domestic and industrial water supply, irrigation, and hydroelectric power purposes, tourism, conservation, and other related activities. This is a multipurpose project aimed at providing socioeconomic development opportunities for the Eastern Cape province [12]. According to the Department of Water and Sanitation [12], the proposed Ntabelanga dam has a storage capacity of 490 million m^3^ and is estimated to supply potable water to 730,000 people by the year 2050. The dam will also provide water to irrigate approximately 2,900 ha of arable land and there will be a small hydropower plant at the dam to generate between 0.75 MW and 5 MW (average 2.1 MW) [12]. Research by Van Tol et al. [13] revealed that large dams play an important role in rejuvenating economic and social development but are often associated with environmental degradation through permanent inundation of previously dry areas, alteration of stream flow regimes, reduction in natural flooding, and fragmentation of river ecosystems, thereby reducing species diversity. The present study reports on plant diversity and useful plant species in the Ntabelanga dam catchment area that will be impacted by the construction of the dam. Results of this study are of interest to the scientific community interested in the uses and values of plant diversity to local communities and associated TEK in the context of large-scale socioeconomic developmental projects.

## 2. Methods

### 2.1. Study Area

**Figure 1:**
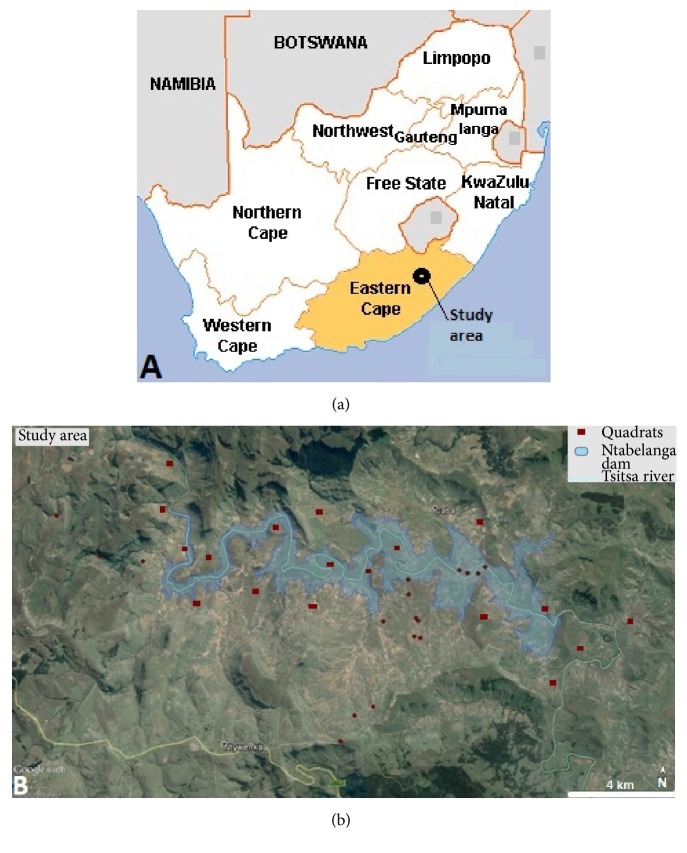
Geographical location of the study area. (a) Map of South Africa illustrating the geographical position of Ntabelanga dam in the Eastern Cape province and (b) detailed map showing position of quadrats along the Tsitsa river.

The Ntabelanga dam catchment area (Figure 1) falls within the semiarid area of the former Transkei homeland in the Eastern Cape province. Large parts of the Eastern Cape province are made up of former homelands of the Apartheid period, namely, Transkei and Ciskei. One of the Apartheid government's acts of segregation was the Bantu Authorities Act of 1951, which legalized the deportation of Black people into designated homelands. Black people were forcibly removed from urban areas and white farms to those areas demarcated as homelands, and such areas are still to a large degree characterized by low capital, poor infrastructure, high unemployment, and high population densities [14]. As a result of this act, Transkei was created in 1951 and the Ciskei in 1961 [15]. According to Hamann and Tuinder [15], the Transkei became the first homeland to be granted the status of “self-governing territory” within the Republic of South Africa in 1963, with the Ciskei homeland following suit in 1972. The Transkei and Ciskei are today characterized by pervasive chronic poverty, low levels of economic activity, dearth of employment opportunities, and high levels of dependency on welfare [16]. An estimated 72% of the population in the Eastern Cape province lives below the poverty line, which is more than the national average of 60% and this is attributed to the legacies of Apartheid, where the Eastern Cape provincial administration inherited the largely impoverished and corrupt former Transkei and Ciskei homelands [17].

Ntabelanga catchment area receives an annual rainfall of about 749 mm, with most of it falling in December and January [18]. The lowest (15 mm) average rainfall is received in June and the highest (108 mm) in January [18]. The area is underlain by sedimentary rocks of the Tarkastad subgroup and Beaufort karoo supergroup with post-karoo doleritic intrusions [18, 19]. The area is characterized by highly unstable soils that are prone to erosion, as evidenced by extensive areas of severe gully erosion on the interfluvial areas adjacent to stream channels [13]. Mucina and Rutherford [20] described the vegetation of the study area as subescarpment grassland and subescarpment savanna bioregions dominated by moist grasslands and* Acacia* spp. This vegetation type occurs at an altitude of 880–1860 m above sea level with the landscape characterized by moderately rolling hills [20]. Households in Ntemalanga catchment area have small permanent arable land between 0.1 ha and 0.5 ha of the 1 ha homestead land allocated to them by the tribal authorities to subsistence agriculture [13]. The arable lands are typically consolidated rainfed farming areas, which can be made up of several plots (1 to 3 ha or more). With poverty, low levels of economic activity, and the poor quality land allocated to Ntabelanga catchment area residents, nonfarm activities are potentially an important source of livelihood.

### 2.2. Data Collection

Plant diversity within the Ntabelanga catchment area was inventoried in March 2016, November 2016, and February 2017. A total of 55 randomly selected quadrats covering potential areas to be impacted by Ntabelanga dam were used to assess plant species diversity and composition (Figure 1(b)). Plant species were identified in the field and the taxon names conform to those of Germishuizen et al. [21]. Unknown plant species were collected, pressed, oven-dried, and identified by taxonomists at the Giffen Herbarium (UFH) at the University of Fort Hare and Schonland Herbarium (GRA) at Rhodes University, Grahamstown, South Africa.

Participatory rural appraisal (PRA) methods [22] were used to identify useful plant species growing in the Ntabelanga catchment area. One hundred and eight randomly selected individuals were interviewed between March 2016 and February 2017, emphasizing in-depth discussions with participants using open-ended questions in data gathering. The questionnaire was administered to one family member, female or male head of the household, or, in the absence of both, any member of the family who was above 18 years. The majority of participants (64.8%) were female and age range of participants was from 19 to 81 years. Structured and semistructured interviews were carried out in isiXhosa, a language spoken by all participants. In order to ensure that participant's right to voluntarily decide to participate in this research on home gardens, all participants were requested to sign the University of Fort Hare (MAR011) consent form, after the researcher or research assistants had fully explained the nature of research work, acknowledged indigenous prior rights and responsibilities of participants, and agreed on active community participation in all stages of the research. The researchers also agreed to a working relationship with the community, including knowledge of and willingness to comply with local governance systems, cultural laws and protocols, social customs, and etiquette as stipulated by the International Society of Ethnobiology (http://www.ethnobiology.net/).

During the interviews, we documented information on

1. names of useful plant species, including species grown and managed in home gardens;
2. uses and preparation of useful plant species;
3. perceptions of households on the importance of plant resources within Ntabelanga catchment area;
4. possible positive and negative impacts of the proposed Ntabelanga dam on availability and utilization of useful plant species that will be assessed based on perceptions of the households.

Results obtained through the use of the questionnaires and PRA exercises were complemented by personal observation, informal discussions, and guided field walks or surveys with the participants.

## 3. Results and Discussion

### 3.1. Plant Use and Taxonomic Diversity

**Figure 2:**
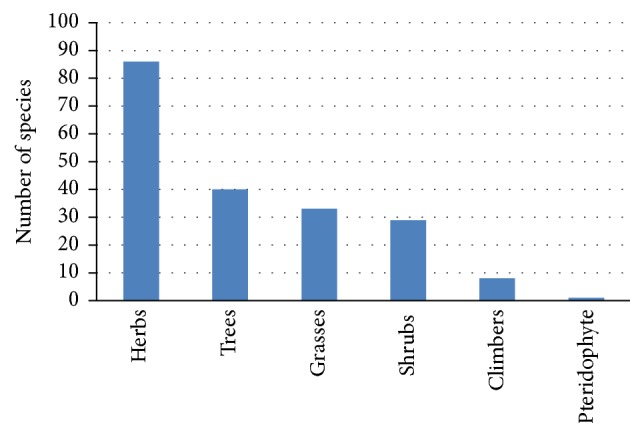
Growth forms observed in Ntabelanga catchment area.

A total of 197 plant species were recorded in the Ntabelanga catchment area (Table 1) with herbs, trees, grasses, and shrubs having the most species (Figure 2). Pteridophyte was represented by a single species,* Cheilanthes hirta *Sw. (family Pteridaceae), while gymnosperm was represented by two species* Podocarpus falcatus *(Thunb.) R. Br. ex Mirb. and* Podocarpus latifolius *(Thunb.) R. Br. ex Mirb. (family Podocarpaceae). Among 197 species recorded in Ntabelanga catchment area, 95 species (48.2%) were utilized for various purposes by the local people (Table 1). About a quarter of these species (28.4%) recorded in Ntabelanga catchment area are exotic to South Africa. Twelve species (6.1%) are declared weeds and invaders in South Africa, listed under the Conservation of Agricultural Resources Act (1983) Number 43 of 1983:* Acacia baileyana* F. Muell.,* Acacia dealbata* Link.,* Acacia mearnsii* De Wild.,* Agave americana* L.,* Catharanthus roseus* (L.) G. Don*, Eucalyptus camaldulensis *Dehnh.,* Eucalyptus grandis *W. Hill ex Maiden,* Melia azedarach *L.,* Opuntia ficus-indica* (L.) Mill.,* Pennisetum clandestinum *Hochst. ex Chiov.,* Psidium guajava* L., and* Ricinus communis *L. [23]. A large number (68.0%, *n* = 134) of the plant species recorded in Ntabelanga catchment area are from 19 families (Table 2). The other 46 families had less representation, between one and two species each. Plant families with the highest number of species were Poaceae (32 species); Asteraceae (26); Fabaceae (12); Cyperaceae (10); Crassulaceae and Solanaceae (five species each); Apiaceae, Asphodelaceae, Celastraceae, Rosaceae, and Rubiaceae (four species each); Amaranthaceae, Anacardiaceae, Apocynaceae, Lamiaceae, Myrtaceae, Polygonaceae, Sterculiaceae, and Vitaceae (three species each) (Table 2). All these plant families with the exception of Celastraceae are among the largest plant families in South Africa, characterized by more than 100 species each [21].

### 3.2. Major Use Categories

**Figure 3:**
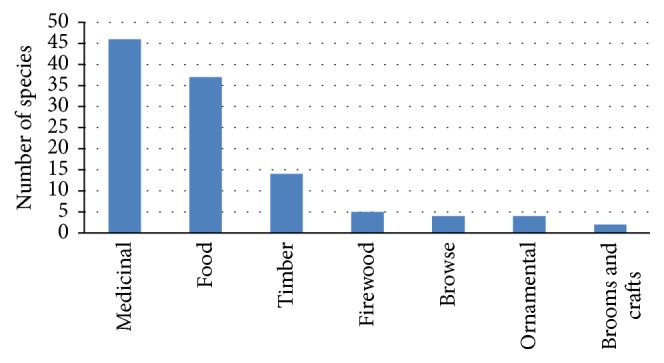
Plant use categories recorded in Ntabelanga catchment area.

Seven major use categories were identified in this study (Table 1, Figure 3), namely, ethnoveterinary and herbal medicines (46 species), food plants (37 species), construction timber and thatching (14 species), firewood (five species), browse, live fence, and ornamental (four species each), and brooms and crafts (two species).

#### 3.2.1. Medicinal Plants

Medicinal plants constituted 46 species and the most important families were Asphodelaceae represented by four species and Apiaceae, Apocynaceae, Asteraceae and Fabaceae represented by three species each. The medicinal plants consisted of mainly herbs (16 species), followed by shrubs (13 species) and trees (11 species). Although the following species are recognized as herbal medicines, they also have other applications:* Acacia karroo* (browse, firewood),* Agave americana* (live fence),* Catharanthus roseus* (ornamental),* Centella coriacea* Nannf. (leafy vegetable),* Citrus limon* (L.) Burm. f. (edible fruits),* Eucalyptus camaldulensis* (construction timber, firewood),* Opuntia ficus-indica* (edible fruits, live fence),* Psidium guajava* (edible fruits),* Schotia latifolia *Jacq. (construction timber), and* Typha capensis* (Rohrb.) N. E. Br. (crafts). Some of the medicinal plants recorded in this study are highly valued medicinal plants in South Africa with potential in the development of new medicinal products with commercial value [24, 25]:* Alepidea amatymbica* Eckl. & Zeyh.,* Aloe arborescens* Mill.,* Aloe ferox* Mill.,* Aloe marlothii* A. Berger,* Carpobrotus edulis *(L.) L. Bolus,* Elephantorrhiza elephantina* (Burch.) Skeels,* Helichrysum nudifolium* (L.) Less.,* Helichrysum odoratissimum* (L.) Sweet,* Hypoxis hemerocallidea* Fisch. & Avé-Lall.,* Leonotis leonurus* (L.) R. Br.,* Pittosporum viridiflorum* Sims,* Typha capensis* (Rohrb.) N. E. Br.,* Xysmalobium undulatum *(L.) W. T. Aiton, and* Ziziphus mucronata* Willd. Previous research by Dold and Cocks [26] revealed that* Alepidea amatymbica*,* Bowiea volubilis *Harv. ex Hook. f. ssp.* volubilis, Bulbine abyssinica *A. Rich.,* Elephantorrhiza elephantina*,* Helichrysum odoratissimum, Hypoxis hemerocallidea, Ilex mitis *(L.) Radlk*., Rhoicissus digitata *(L.f.) Gilg & Brandt, and* Xysmalobium undulatum* are heavily harvested for the medicinal plant trade in the Eastern Cape province. The IUCN Red List Categories and Criteria version 3.1 of threatened species (http://www.iucnredlist.org/) was used by Raimondo et al. [27] to assess the conservation status of* Alepidea amatymbica* and* Bowiea volubilis* ssp.* volubilis* categorizing them as Endangered (A2d) and Vulnerable (VUA2ad), respectively, as the two species are overexploited for traditional medicine trade. According to Victor and Keith [28] and von Staden et al. [29], a species categorized as Least Concern (LC) under the IUCN Red List Categories and Criteria version 3.1 can additionally be flagged as of conservation concern either as rare, critically rare, or declining; hence* Hypoxis hemerocallidea *and* Ilex mitis *are categorized as declining by Raimondo et al. [27]. Some of these species, including* Alepidea amatymbica, Artemisia afra, Bowiea volubilis* ssp.* volubilis, Catharanthus roseus*, and* Tulbaghia acutiloba *were cultivated in home gardens in Ntabelanga catchment area mainly due to scarcity and high demand for the species. Previous research by Wiersum et al. [30] also found* Alepidea amatymbica *and* Bowiea volubilis* ssp.* volubilis* to be some of the preferred medicinal plants that are cultivated in home gardens in the Eastern Cape province as herbal medicines. Wiersum et al. [30] argued that cultivation of medicinal plants can serve as a tool for combined biodiversity conservation and poverty alleviation, resulting in increased social capital and human dignity. Therefore, Ntabelanga catchment area harbours some important medicinal plant species which have an important contribution to primary health care and are source of income through trade and cultural heritage of the local people.

PRA exercises with participants and observations made on main livelihood attributes in the study area seem to suggest that the TEK, practices and beliefs of the Xhosa people, are dynamic and adaptive. This can be seen in the incorporation of exotic plant species to South Africa in the indigenous pharmacopoeia of the Ntabelanga catchment area residents. Exotic plants which are now part of the indigenous pharmacopoeia in the study area include* Agave americana*,* Catharanthus roseus, Ficus carica, Opuntia ficus-indica, Psidium guajava*, and* Sonchus asper.* Palmer [31] argued that the medicinal plant composition of a community is the product of experimentations conducted throughout the history of a community and represents an adaptation of this culture over time. While Alencar et al. [32] argued that any indigenous medical system is not a static social institution that is not evolving, as there is evidence of insertions and deletions of plants that compose it, with the addition of exotic plants as herbal medicines. Therefore, results of the current study corroborate an earlier observation that TEK systems are a reservoir of experiential knowledge that can provide important insights for the design of adaptation and mitigation strategies to cope with global environmental change [33].

#### 3.2.2. Food Plants

A variety of food plants were recorded in Ntabelanga catchment area, mainly edible fruits (19 species) and leafy vegetables (11 species) and edible bulbs, roots, or tubers (6 species). Based on PRA exercises,* Zea mays *L. was among the most important food plants, grown as a cereal or beverage, with its dry seeds pounded into samp or green mealies either roasted or cooked. The most represented families were Asteraceae represented by 5 species and Rosaceae and Solanaceae with 4 species each. The majority of food plants were herbs (19 species), trees (eight species), and five climbers. The majority of food plants (91.9%) were exotic to South Africa, only* Carissa bispinosa *(L.) Desf. ex Brenan and* Dovyalis caffra* (Hook. f. & Harv.) Hook. f., both classified as edible fruits, and, a leafy vegetable,* Centella coriacea *Nannf. are indigenous. Some food plants such as* Citrus limon*,* Opuntia ficus-indica*, and* Psidium guajava *were also used as herbal medicines. Important food plants mentioned by more than 25 percent of the participants included* Allium cepa *L. (onion),* Brassica oleracea *L. (cabbage),* Capsicum annuum *L. (pepper),* Citrus limon *(lemon),* Citrus sinensis *(L.) Osbeck (orange),* Cucurbita maxima *Duchesne (pumpkin),* Cucurbita moschata *Duchesne ex Poir. (butternut),* Daucus carota *L. (carrot),* Lycopersicon esculentum *Mill. (tomato),* Solanum tuberosum *(potato),* Spinacia oleracea *(spinach), and* Zea mays *(maize). The diversity of food plants documented in this study indicates the relevance of the Ntabelanga catchment area as an important resource for food production and subsistence of households. One of the main purposes of agricultural environments of traditional communities is to produce food and the high agrobiodiversity found in these areas increases the nutritional diversity and quality of family diets [34].

#### 3.2.3. Other Plant Use Categories

Residents in Ntabelanga catchment area used species such as* Acacia baileyana*,* Acacia caffra* (Thunb.) Wild.,* Acacia dealbata*,* Acacia mearnsii*,* Bruguiera gymnorrhiza* (L.) Lam.,* Eucalyptus camaldulensis*,* Eucalyptus grandis*, and* Schotia latifolia* Jacq. to construct huts, fence, and different types of enclosures. Grass species which included* Cymbopogon nardus* (L.) Rendle,* Hyparrhenia hirta* (L.) Stapf,* Miscanthus capensis* (Nees) Andersson,* Phragmites australis* (Cav.) Steud.,* Sporobolus africanus* (Poir.) Robyns & Tournay, and* Sporobolus fimbriatus* (Trin.) Nees were harvested to thatch traditional structures such as huts and enclosures. Five species, namely,* Acacia dealbata*,* Acacia karroo*,* Acacia mearnsii*,* Eucalyptus camaldulensis*, and* Eucalyptus grandis*, were used as fuel wood and for space heating.* Agave americana*,* Catharanthus roseus*,* Opuntia ficus-indica*, and* Phoenix reclinata* Jacq. were cultivated as live fence and ornamental plants.* Phoenix reclinata* and* Typha capensis* were used for making crafts such mats and baskets.* Phoenix reclinata* leaves were shredded and bound together to make brooms. Leaves of* Acacia karroo*,* Combretum erythrophyllum* (Burch.) Sond.,* Cynodon dactylon* (L.) Pers., and* Diospyros lycioides* Desf. were browsed by livestock, mainly cattle and goats.

### 3.3. Perceptions of Participants Regarding Dam Construction

The value of plant resources as a source of household livelihoods needs was ubiquitously perceived, with majority of participants reporting negative impacts likely to be caused by the planned Ntabelanga dam (Table 3). The majority of participants (58.3%) revealed that the planned dam will negatively affect the availability of edible, medicinal, and other useful plants currently collected from the wild within the Ntabelanga catchment area. Such sentiments echoed by the residents are supported by the literature as flooding upstream of dams results in the permanent destruction of terrestrial ecosystems through inundation and all terrestrial plants and animals disappear from the submerged area [35]. Other social impacts associated with new dams include enforced displacement of populations, migration, social disruption, loss of habitats, loss of biodiversity, loss of access to resources, loss of cultural capital, and the depletion of natural resources [35].

About 15.7% of the participants are convinced that the proposed dam will reduce the grazing land, leading to overstocking and overgrazing. The PRA exercises with the participants revealed that reduction of grazing land through damming will cause carrying capacity to diminish over time and, therefore, the quality and productivity of livestock will deteriorate through lower calving rates and lower annual growth of individual species. According to participants, another consequence of damming is that total biomass will be reduced, leading to overgrazing of the available grazing lands. This means that more pressure will be placed on remaining grasslands and the process will accelerate over time, leading to runaway erosion and further loss of palatable grass species. Damming will force residents to graze their livestock in residential areas, cropping land, and abandoned or old cropping lands due to reduced grazing land. Our assessment revealed that abandoned old cropping lands usually have annual weedy species and other related species which are of limited grazing value. Livestock farming is important to Ntabelanga residents and the greater part of the Eastern Cape province which contributes over a third of South Africa's livestock species, about 35% for cattle, 57% for goats, and 10% for sheep [36]. Interviews with participants revealed that livestock are considered to be an important status symbol of residents in Ntabelanga catchment area and also provide ready cash to the household through sales when the need arises. Cattle are used in paying bride prizes, while goats and sheep are mainly used for traditional and religious sacrifices. Cousins [37] and Mmbengwa et al. [36] argue that livestock, particularly cattle, form a fundamental part of the lives of rural people's lifestyle in South Africa, as cattle are often used in paying lobola (bride's worth) and other social activities.

According to 7.4% of the participants (Table 3), construction of Ntabelanga dam will cause an increase in the number of alien plant species, weeds, pests, and diseases. From literature, biodiversity changes are expected as a result of damming and new dams may increase susceptibility to species invasion in a number of ways [38]. These authors also argue that the introduction of exotic species or changes in community composition can affect ecosystem goods and services either by directly reducing abundances of useful species or by altering controls on critical ecosystem processes. Damming is also known to pose potential threat to public health as dams tend to harbour a wide diversity of water-associated pathogens such as mosquito vectors carrying malaria, schistosomiasis, diarrhea, and dysentery causing protozoa [39]. Therefore, damming could potentially trigger an increase in the incidence of many of these neglected tropical diseases, because their epidemiology is inherently linked to wetland ecology and accumulation of surface water [39]. Some participants preferred to emphasize the social and economic benefits associated with damming, focusing on ecosystem goods and services that will be positively affected by the Ntabelanga dam. PRA exercises revealed that some participants (11.1%) foresee an increase in plant diversity cultivated in home gardens, with a possibility of year-round production due to enhanced water supply (Table 3). According to some participants (10.2%), availability of water in home gardens is one of the essential resources required to ensure food production in the Ntabelanga catchment area. Previous research by McCartney et al. [35] revealed that many large dams provide irrigation services that benefit millions of people throughout the world in terms of increased output of food, new livelihood opportunities such as tourism, transportation, and increased marketing of produce to industrial populations, which can increase produce prices and thus farm incomes.

## 4. Conclusion

The construction of Ntabelanga dam is associated with several positive and negative impacts on plant resources which are not fully integrated into current decision-making largely because of lack of multistakeholder dialogue on the socioeconomic issues of such an important project. The PRA exercises revealed that the socioeconomic environment associated with Ntabelanga dam catchment area is very diverse, characterized by wide ranging and sometimes contradicting livelihood needs mostly centred on ecosystem services and goods provided by plant resources. Based on these PRA exercises, it is concluded that any major socioeconomic development project such as Ntabelanga dam should be part of a larger framework aimed at addressing poverty, power, and inequalities that particularly affect the poorest members of the community. The challenge of providing long-term access to and protecting and managing ecosystem services for the poorest and most vulnerable people depending on those services appears not to have been adequately addressed. There is need to stimulate multistakeholder dialogue on ways to review the tools and approaches currently in use around the world to better share the benefits that arise from using such large dams. It is also important to ensure that any households to be displaced by the proposed Ntabelanga dam will benefit directly from the development opportunities generated by the proposed project in order to improve their living standards. The PRA exercises revealed that the majority of participants want Ntabelanga dam operations and management to promote agricultural and off-farm activities that promote functioning and diversity of ecosystem services that enhance food security and agricultural production. These regulating, supporting, and cultural services usually provide the fundamental basis for local livelihoods and well-being and sometimes directly lift the poorest and marginalized households out of poverty.

## Figures and Tables

**Table 1:** Plant species recorded in Ntabelanga catchment area in the Eastern Cape province, South Africa. Species marked with asterisk (*∗*) are exotic to South Africa.

Species and family name	Family	Growth form	Uses^#^						
			B	C	F	M	O	T	V
^*∗*^Acacia baileyana F. Muell.	Fabaceae	Tree						X	
Acacia caffra (Thunb.) Wild.	Fabaceae	Tree						X	
^*∗*^Acacia dealbata Link.	Fabaceae	Tree			X			X	
Acacia karroo Hayne	Fabaceae	Tree	X		X	X			
^*∗*^Acacia mearnsii De Wild.	Fabaceae	Tree			X			X	
*Acokanthera oblongifolia *(Hochst.) Codd	Apocynaceae	Tree				X			
Agathisanthemum bojeri Klotzsch	Rubiaceae	Herb							
^*∗*^ *Agave americana *L.	Asparagaceae	Shrub				X	X		
*Alepidea amatymbica *Eckl. & Zeyh.	Apiaceae	Herb				X			
*Alepidea serrata *Eckl. & Zeyh.	Apiaceae	Herb				X			
^*∗*^ *Allium cepa *L.	Alliaceae	Herb							X
^*∗*^ *Allium sativum *L.	Alliaceae	Herb							X
*Aloe arborescens* Mill.	Asphodelaceae	Shrub				X			
*Aloe ciliaris *Haw.	Asphodelaceae	Shrub				X			
*Aloe ferox *Mill.	Asphodelaceae	Shrub				X			
*Aloe marlothii* A. Berger	Asphodelaceae	Tree				X			
^*∗*^ *Amaranthus hybridus *L.	Amaranthaceae	Herb							X
*Andropogon eucomus *Nees	Poaceae	Grass							
*Anthospermum galioides* Rchb. f.	Rubiaceae	Herb							
*Aristida congesta *Roem. & Schult.	Poaceae	Grass							
*Artemisia afra *Jacq. ex Willd.	Asteraceae	Shrub				X			
*Arundinella nepalensis *Trin.	Poaceae	Grass							
*Asparagus asparagoides *(L.) Druce	Asparagaceae	Climber				X			
*Asparagus laricinus *Burch.	Asparagaceae	Shrub				X			
*Berkheya bergiana *Söderb.	Asteraceae	Herb							
*Berkheya discolor* (DC.) O. Hoffm. & Muschl.	Asteraceae	Herb							
*Berkheya bipinnatifida* (Harv.) Roessler	Asteraceae	Herb							
^*∗*^ *Beta vulgaris *L.	Chenopodiaceae	Herb							X
^*∗*^ *Bidens pilosa *L.	Asteraceae	Herb							X
*Bowiea volubilis *Harv. ex Hook. f. ssp *volubilis*	Hyacinthaceae	Herb				X			
^*∗*^ *Brassica oleracea *L.	Brassicaceae	Herb							X
^*∗*^ *Brassica rapa *L.	Brassicaceae	Herb							X
*Bruguiera gymnorrhiza *(L.) Lam.	Rhizophoraceae	Tree						X	
*Buddleja saligna *Willd.	Buddlejaceae	Tree							
*Bulbine abyssinica *A. Rich.	Xanthorrhoeaceae	Herb				X			
*Bulbostylis contexta* (Nees) Bodard	Cyperaceae	Herb							
*Bulbostylis densa* (Wall.) Hand.-Mazz.	Cyperaceae	Herb							
*Bulbostylis hispidula *(Vahl) R. W. Haines	Cyperaceae	Herb							
*Capparis tomentosa *Lam.	Capparaceae	Tree				X			
^*∗*^ *Capsicum annuum *L.	Solanaceae	Herb							X
*Carissa bispinosa *(L.) Desf. ex Brenan	Apocynaceae	Shrub							X
*Carpobrotus edulis *(L.) L. Bolus	Mesembryanthemaceae	Shrub				X			
^*∗*^ *Catharanthus roseus *(L.) G. Don	Apocynaceae	Herb				X	X		
*Celtis africana* Burm. f.	Celastraceae	Shrub							
*Centella coriacea *Nannf.	Apiaceae	Herb				X			X
*Chaenostoma campanulatum *Benth.	Scrophulariaceae	Herb							
*Chamaecrista capensis* (Thunb.) E. Mey.	Fabaceae	Herb							
^*∗*^Chenopodium album L.	Chenopodiaceae	Herb							X
*Cheilanthes hirta *Sw.	Pteridaceae	Pteridophyte				X			
*Chloris virgata *Sw.	Poaceae	Grass							
^*∗*^ *Citrus limon *(L.) Burm. f.	Rutaceae	Tree				X			X
^*∗*^ *Citrus sinensis *(L.) Osbeck	Rutaceae	Tree							X
*Combretum erythrophyllum *(Burch.) Sond.	Combretaceae	Tree	X						
*Commelina africana *L.	Commelinaceae	Herb							
*Convolvulus sagitattarius *Thumb	Convolvulaceae	Herb				X			
^*∗*^ *Conyza bonariensis *(L.) Cronquist	Asteraceae	Herb							
*Conyza pinnata* (L. f.) Kuntze	Asteraceae	Herb							
*Crabbea hirsuta* Harv.	Acanthaceae	Herb							
*Crassula ericoides* Haw.	Crassulaceae	Shrub							
*Crassula nudicaulis *L.	Crassulaceae	Shrub							
*Crassula setulosa* Harv.	Crassulaceae	Shrub							
^*∗*^ *Cucurbita maxima *Duchesne	Cucurbitaceae	Climber							X
^*∗*^ *Cucurbita moschata *Duchesne ex Poir.	Cucurbitaceae	Climber							X
*Cussonia paniculata* Eckl. & Zeyh.	Araliaceae	Tree							
*Cussonia spicata *Thunb.	Araliaceae	Tree				X			
*Cymbopogon nardus* (L.) Rendle	Poaceae	Grass						X	
^*∗*^ *Cynodon dactylon* (L.) Pers.	Poaceae	Grass	X						
*Cyperus albostriatus *Schrad.	Cyperaceae	Herb							
*Cyperus brevis* Boeck.	Cyperaceae	Herb							
*Cyperus congestus* Vahl	Cyperaceae	Herb							
*Cyphostemma setosum* (Roxb.) Alston	Vitaceae	Climber							
*Dactyloctenium giganteum *Fisher & Schweick.	Poaceae	Grass							
^*∗*^ *Daucas carota *L.	Apiaceae	Herb							X
*Digitaria ternata* (A. Rich.) Stapf	Poaceae	Grass							
*Diospyros austro-africana *De Winter	Ebenaceae	Shrub							
*Diospyros lycioides *Desf.	Ebenaceae	Shrub	X						
*Dovyalis caffra* (Hook. f. & Harv.) Hook. f.	Flacourtiaceae	Shrub							X
*Elephantorrhiza elephantina *(Burch.) Skeels	Fabaceae	Shrub				X			
*Eragrostis chloromelas* Steud.	Poaceae	Grass							
*Eragrotis curvula* (Schrad.) Nees	Poaceae	Grass							
*Eragrostis gummiflua* Nees	Poaceae	Grass							
*Eragrostis plana* Nees	Poaceae	Grass							
*Eragrostis racemosa* (Thunb.) Steud.	Poaceae	Grass							
^*∗*^ *Eucalyptus camaldulensis *Dehnh.	Myrtaceae	Tree			X	X		X	
^*∗*^ *Eucalyptus grandis *W. Hill ex Maiden	Myrtaceae	Tree			X			X	
*Euphorbia inaqualatera* Sond.	Euphorbiaceae	Shrub							
*Finicia brevifolia* Kunth.	Cyperaceae	Herb							
*Ficinia deusta *(P. J.) Bergius) Levyns	Cyperaceae	Herb							
^*∗*^ *Ficus carica *L.	Moraceae	Tree							X
*Flacourtia indica *(Burm. f.) Merr.	Flacourtiaceae	Shrub							
*Gomphrena celosioides* Mart.	Amaranthaceae	Herb							
*Gymnosporia buxifolia* (L.) Szyszyl.	Celastraceae	Tree							
*Gymnosporia harveyana *Loes.	Celastraceae	Tree							
*Gymnosporia nemerosa *(Eckl. & Zeyh.) Szyszyl.	Celastraceae	Tree							
*Gymnosporia senegalensis *(Lam.) Loes.	Celastraceae	Tree							
*Harpochloa falx* (L. f.) Kuntze61	Poaceae	Grass							
*Helichrysum herbaceum *(Andrews) Sweet	Asteraceae	Herb							
*Helichrysum glomeratum* Klatt	Asteraceae	Herb							
*Helichrysum gymnocomum *DC.	Asteraceae	Herb				X			
*Helichrysum krebsianum *Less.	Asteraceae	Herb							
*Helichrysum nudifolium* (L.) Less.	Asteraceae	Herb				X			
*Helichrysum odoratissimum* (L.) Sweet	Asteraceae	Shrub				X			
*Helichrysum oreophilum* Klatt	Asteraceae	Herb							
*Hermannia depressa* N. E. Br.	Sterculiaceae	Herb							
*Hermannia parviflora* Eckl. & Zeyh.	Sterculiaceae	Herb							
*Hermannia transvaalensis *Schinz	Sterculiaceae	Herb							
*Heteropogon contortus *(L.) Roem. & Schult.	Poaceae	Grass							
*Hyparrhenia hirta* (L.) Stapf	Poaceae	Grass						X	
*Hyparrhenia tamba *(Steud.) Stapf	Poaceae	Grass							
*Hypoestes forskaolii* (Vahl) R. Br.	Acanthaceae	Herb							
*Hypoxis argentea* Harv. ex Baker	Hypoxidaceae	Herb				X			
*Hypoxis hemerocallidea* Fisch. Mey. & Ave-Lall.	Hypoxidaceae	Herb				X			
*Ilex mitis *(L.) Radlk.	Aquifoliaceae	Tree				X			
*Imperata cylindrica *(L.) Raeusch.	Poaceae	Grass							
^*∗*^ *Ipomoea batatas *(L.) Lam.	Convolvulaceae	Climber							X
*Kalanchoe rotundifolia* (Paw.) Paw.	Crassulaceae	Shrub							
*Kalanchoe thyrsiflora* Harv.	Crassulaceae	Shrub							
*Kyllinga alata* Nees	Cyperaceae	Herb							
^*∗*^ *Lactuca sativa* L.	Asteraceae	Herb							X
*Lantana rugosa* Thunb.	Verbenaceae	Shrub							
*Leersia hexandra *Sw.	Poaceae	Grass							
*Leonotis leonurus *(L.) R. Br.	Lamiaceae	Shrub				X			
*Leucosidea sericea* Eckl. & Zeyh.	Rosaceae	Shrub							
*Lobelia flaccida* (C. Presl) A. DC.	Campanulaceae	Herb				X			
*Lobelia thermalis* Thunb.	Campanulaceae	Herb							
^*∗*^ *Lycopersicon esculentum *Mill.	Solanaceae	Climber							X
^*∗*^ *Malus domestica *Borkh.81	Rosaceae	Tree							X
^*∗*^ *Melia azedarach *L.	Meliaceae	Tree							
*Melinis nerviglumis* (Franch.) Zizka	Poaceae	Grass							
*Melinis repens* (Willd) Zizka	Poaceae	Grass							
*Microchlon caffra *Nees	Poaceae	Grass							
*Miscanthus capensis *(Nees) Andersson	Poaceae	Grass						X	
*Miscanthus junceus *(Stapf) Pilg.	Poaceae	Grass							
^*∗*^ *Musa* X *paradisiaca *L.86	Musaceae	Tree							X
^*∗*^ *Nicotiana glauca *Graham87	Solanaceae	Shrub							
*Nidorella pinnata *(L. f.) J.C. Manning & Goldbalt	Asteraceae	Herb							
^*∗*^ *Oenothera rosea *L'Hér. ex Aiton	Onagraceae	Herb							
^*∗*^ *Opuntia ficus-indica *(L.) Mill.	Cactaceae	Tree				X	X		X
*Oxalis smithiana* Eckl. & Zeyh.	Oxalidaceae	Herb							
*Panicum maximum *Jacq.	Poaceae	Grass							
^*∗*^ *Paspalum distichum* L.	Poaceae	Grass							
^*∗*^ *Pennisetum clandestinum *Hochst. ex Chiov.	Poaceae	Grass							
^*∗*^ *Persea americana *Mill.	Lauraceae	Tree							X
*Persicaria attenuata *(R. Br.) Soják	Polygonaceae	Herb							
*Persicaria decipiens *(R. Br.) Wilson	Polygonaceae	Herb							
^*∗*^ *Phaseolus vulgaris *L.	Fabaceae	Herb							X
*Phoenix reclinata *Jacq.	Arecaceae	Tree		X			X		
*Phragmites australis *(Cav.) Steud.	Poaceae	Grass						X	
^*∗*^ *Pisum sativum *L.	Fabaceae	Herb							X
*Pittosporum viridiflorum *Sims.	Pittosporaceae	Shrub				X			
^*∗*^ *Plantago lanceolata *L.	Plantaginaceae	Herb							
*Podocarpus falcatus *(Thunb.) R. Br. ex Mirb.	Podocarpaceae	Tree							
*Podocarpus latifolius *(Thunb.) R. Br. ex Mirb.	Podocarpaceae	Tree							
*Polygala amatymbica* Eckl. & Zeyh.	Polygalaceae	Herb							
^*∗*^ *Prunus armeniaca *L.	Rosaceae	Tree							X
^*∗*^ *Prunus persica *(L.) Batsch	Rosaceae	Tree							X
^*∗*^ *Psidium guajava *L.	Myrtaceae	Shrub				X			X
*Rhoicissus digitata *(L.f.) Gilg & Brandt	Vitaceae	Climber				X			
^*∗*^ *Richardia brasiliensis* Gomes	Rubiaceae	Herb							
^*∗*^ *Richardia humistrata* (Cham. & Schltdl.) Steud.	Rubiaceae	Herb							
^*∗*^ *Ricinus communis *L.	Euphorbiaceae	Tree				X			
^*∗*^ *Salix babylonica *L.	Salicaceae	Tree							
*Salvia scabra *L. f.	Lamiaceae	Herb				X			
^*∗*^ *Schkuhria pinnata* (Lam.) Cabrera	Asteraceae	Herb							
*Schoenoplectus brachycerus *(A. Rich.) Lye	Cyperaceae	Herb							
*Schoenoplectus corymbosus *(Roem. & Schult.) J. Raynal	Cyperaceae	Herb							
*Schotia latifolia *Jacq.	Fabaceae	Tree				X		X	
*Searsia dentata* (Thunb.) F. A. Barkley	Anacardiaceae	Tree							
*Searsia pentheri* (Zahlbr.) Moffett	Anacardiaceae	Tree							
*Searsia pyroides *(Burch.) Moffett	Anacardiaceae	Shrub							
*Senecio decurrens *DC.	Asteraceae	Herb							
*Senecio inaequidens* DC.	Asteraceae	Herb							
*Senecio retrorsus *DC.	Asteraceae	Herb							
*Setaria sphacelata* (Schumach.) Stapf & C.E.Hubb. ex Moss	Poaceae	Grass							
*Sida rhombifolia *L.	Malvaceae	Shrub				X			
*Solanum aculeastrum *Dun.	Solanaceae	Shrub				X			
^*∗*^ *Solanum tuberosum *L.	Solanaceae	Herb							X
^*∗*^ *Sonchus asper *(L.) Hill	Asteraceae	Herb							X
^*∗*^ *Sonchus oleraceus *L.	Asteraceae	Herb							X
^*∗*^ *Spinacia oleracea *L.	Amaranthaceae	Herb							X
*Sporobolus africanus* (Poir.) Robyns & Tournay	Poaceae	Grass		X					
*Sporobolus festivus* Hochst. ex A. Rich.	Poaceae	Grass							
*Sporobolus fimbriatus* (Trin.) Nees	Poaceae	Grass						X	
*Stachys aethiopica* L.	Lamiaceae	Herb							
^*∗*^ *Tagetes minuta *L.	Asteraceae	Herb							
^*∗*^ *Taraxacum officinale* Weber	Asteraceae	Herb							X
*Tephrosia capensis* (Jacq.) Pers.	Fabaceae	Herb							
*Teucrium trifidum* Retz.	Lamiaceae	Herb							
*Trema orientalis* (L.) Blume	Celtidaceae	Tree							
*Tulbaghia acutiloba *Harv.	Alliaceae	Herb				X			
*Typha capensis *(Rohrb.) N. E. Br.	Typhaceae	Herb		X		X			
*Vernonia natalensis* Oliv. & Hiern	Asteraceae	Herb							
^*∗*^ *Vitis vinifer *L.	Vitaceae	Climber							X
*Xysmalobium undulatum (L.) *W.T. Aiton	Apocynaceae	Herb				X			
^*∗*^ *Zea mays *L.	Poaceae	Grass							X
^*∗*^ *Zinnia peruviana *(L.) L.	Asteraceae	Herb							
*Ziziphus mucronata* Willd.	Rhamnaceae	Tree				X			
*Zornia capensis *Pers.	Fabaceae	Herb							

^#^B = browse, C = brooms and crafts, F = firewood, M = medicinal, O = live fence and ornamentals, T = construction timber and thatching, and V = food plants.

**Table 2:** Plant families with the largest number of species (with more than 3 species) in the Ntabelanga catchment area.

Family	Number of species	%
Poaceae	32	16.2
Asteraceae	26	13.2
Fabaceae	12	6.1
Cyperaceae	10	5.1
Crassulaceae	5	2.5
Solanaceae	5	2.5
Apiaceae	4	2.0
Asphodelaceae	4	2.0
Celastraceae	4	2.0
Rosaceae	4	2.0
Rubiaceae	4	2.0
Amaranthaceae	3	1.5
Anacardiaceae	3	1.5
Apocynaceae	3	1.5
Lamiaceae	3	1.5
Myrtaceae	3	1.5
Polygonaceae	3	1.5
Sterculiaceae	3	1.5
Vitaceae	3	1.5

**Table 3:** Perceptions on possible impacts of dam construction on availability of plant resources in Ntabelanga catchment area.

Variable	Proportion (%)
Edible plants and herbal medicines collected from the wild will be negatively affected	58.3
Size of grazing land to be reduced	15.7
It will be possible to have home garden produce throughout the year	13.0
Availability of water will result in increased plant diversity in home gardens	11.1
Availability of water will result in revival of home gardening activities	10.2
Number of problem plants including alien species and weeds, pests and diseases will increase	7.4
